# miR-451 Silencing Inhibited Doxorubicin Exposure-Induced Cardiotoxicity in Mice

**DOI:** 10.1155/2019/1528278

**Published:** 2019-07-04

**Authors:** Jun Li, Weiguo Wan, Tao Chen, Suiyang Tong, Xuejun Jiang, Wanli Liu

**Affiliations:** ^1^Department of Cardiology, Renmin Hospital of Wuhan University, Wuhan, Hubei 430060, China; ^2^Department of Pediatric Cardiovascular Medicine, Woman and Child Hospital of Hubei Province, Wuhan, Hubei, 430060, China

## Abstract

Oxidative stress and cardiomyocytes apoptosis were closely involved in the pathological process of doxorubicin- (Dox-) induced cardiac injury. MicroRNA-451 (miR-451) was mainly expressed in cardiomyocytes. However, the role of miR-451 in Dox-induced cardiac injury remained unclear. Our study aimed to investigate the effect of miR-451 on Dox-induced cardiotoxicity in mice. We established a Dox-induced cardiotoxicity model in the mice and manipulated miR-451 expression in the heart using a miR-451 inhibitor, which was injected every other day beginning at one day before Dox injection. Oxidative stress and apoptosis in the hearts were evaluated. miR-451 levels were significantly increased in Dox-treated mice or cardiomyocytes. miR-451 inhibition attenuated Dox-induced whole-body wasting and heart atrophy, reduced cardiac injury, restored cardiac function, and improved cardiomyocyte contractile function. Moreover, miR-451 inhibition reduced oxidative stress and cardiomyocytes apoptosis in vivo and in vitro. miR-451 inhibition increased the expression of calcium binding protein 39 (Cab39) and activated adenosine monophosphate activated protein kinase (AMPK) signaling pathway. A specific inhibitor of AMPK abolished the protection provided by miR-451 inhibition against cell injury in vitro. In conclusion, miR-451 inhibition protected against Dox-induced cardiotoxicity via activation of AMPK signaling pathway.

## 1. Introduction

Doxorubicin (Dox) is an anthracycline cytostatic agent, which has been commonly used to treat solid tumors, lymphomas, and child leukemias [1]. Dox administration resulted in the arrest of cell cycle and inhibited the proliferation of tumor cells [2]. Dox caused cumulative dose-limiting toxicity, which could result in irreversible degenerative cardiomyopathy and heart failure with long-term heart problems [3]. The mechanism of Dox-induced cardiotoxicity involved the generation of oxidative stress, DNA damage, and cardiomyocytes loss [4, 5]. Currently, there was no effective approach to prevent Dox-related cardiotoxicity. Development of novel interventions that can inhibit these pathological alterations will reduce or prevent this complication related to Dox in patients with tumor.

MicroRNAs (miRNAs, miRs) are highly conserved small noncoding RNAs. miRNAs negatively regulate gene expressions by interacting with the 3'-untranslated region of the target mRNA [6]. Previous reports indicated that miRNAs were closely involved in the process of Dox-related cardiac injury in mice. miR-21 suppression inhibited cardiac alterations induced by Dox [7]. miR-140-5p aggravated Dox-induced cardiotoxicity by promoting oxidative stress in the hearts [8]. A recent study has corroborated the potential role of miR-451 in cardiovascular diseases. Human microRNA-451 (miR-451) gene is located on chromosome 17 at 17q11.2, at the downstream of the miR-144 gene [9]. Among vertebrates, miR-451 is highly conserved. Previous study found that miR-451 expression was mainly restricted to erythropoietic cells [10]. miR-451 inhibition in erythroleukemia cells resulted in the impairment of erythrocyte differentiation [11]. Kuwabara et al. found that miR-451 was also highly expressed in cardiac myocytes, and miR-451 could promote lipotoxicity and cardiac hypertrophy in obese mice [12]. miR-451 directly interacted with the 3'-UTRs of Cab39 [12], which was an armadillo repeat scaffolding-like protein and a component of the trimeric liver kinase B1 (LKB1)-Cab39 complex. Cab39 stabilized the activity of LKB1 and thus increased phosphorylation of adenosine monophosphate activated protein kinase (AMPK) [13, 14].

AMPK, a major cellular sensor of energy availability, is a key regulator of various pathological processes in the heart [15]. A previous study found that activation of AMPK*α* attenuated aortic binding-induced cardiac remodeling in mice [16]. AMPK*α* has emerged as a novel regulator for several cellular actions including cell apoptosis and cell redox reaction [17]. A recent study found that AMPK*α* activation by melatonin exerted protection against acute cardiotoxicity through preservation of mitochondrial homeostasis [18]. Whether miR-451 could attenuate Dox-related cardiac injury via restoring the phosphorylation of AMPK remains unclear. Therefore, the aim of the present study was to investigate the role of miR-451 and the underlying mechanism in mice using a miR-451 inhibitor.

## 2. Materials and Methods

### 2.1. Reagents

Dox (Cat. No: D1515, purity >98%) was obtained from Sigma-Aldrich (St. Louis, MO, USA), which was dissolved in saline for in vivo experiments and in PBS for in vitro tests. Compound C (Dorsomorphin, Cat. No: P5499) was also obtained from Sigma-Aldrich. The protein concentrations were determined using a bicinchoninic acid (BCA) protein assay kit (Bio-Rad, Hercules, CA, USA). The N-terminal pro brain natriuretic peptide (NT-proBNP) ELISA kit was obtained from My BioSource (CA, USA). Cardiac troponin I (cTnI) ELISA kit was obtained from Life Diagnostics, Inc. (West Chester, PA). The creatine kinase (CK) kit was obtained from Nanjing Jiancheng Bioengineering Institute (Nanjing, China). The kits for measuring reduced glutathione (GSH), oxidized glutathione (GSSG) and malondialdehyde (MDA) concentrations, and total superoxide dismutase (SOD) activity were provided by Nanjing Jiancheng Bioengineering Institute. Lipid peroxidation (4-hydroxynonenal, 4-HNE) Assay kit was obtained from Abcam (Cambridge, MA, UK). Anti-AMPK (#50081), anti-phospho-AMPK (p-AMPK, Thr172, #5831), anti-calcium binding protein 39 (Cab39, #ab51132), anti-phospho-mammalian target of rapamycin (p-mTOR, #ab84400), anti-mTOR (#ab134903), anti-phospho-p70 (#ab109393), anti-p70 (#ab184551), and anti-*β*-actin (ab6276) were obtained from Abcam. The miR-451 inhibitor and NC inhibitor were provided by RiboBio (Guangzhou, China)

### 2.2. Animals and Treatments

All animal experiments were cared for humanely in accordance with the Guide for the Care and Use of Laboratory Animals published by the U.S. National Institutes of Health (National Institutes of Health Publication No. 85-23, revised 1996), and all in vivo experiment protocols were approved by the Ethical Committee of Renmin Hospital of Wuhan University and Woman and Child Hospital of Hubei Province. Healthy adult male C57BL/6 mice (25±2g, 9-11 weeks) were provided by HFK Bioscience (Beijing, China). Forty mice were randomly assigned to four groups (n=10 for each group): negative control (NC) inhibitor+saline, miR-451 inhibitor+saline, NC inhibitor+Dox, miR-451 inhibitor+Dox. To mimic Dox exposure, the mice in the NC inhibitor+Dox and miR-451 inhibitor+Dox groups were intraperitoneally injected with 15 mg/kg of Dox according to a previous study [1]. The mice in the NC inhibitor+saline and miR-451 inhibitor+saline received the same volume of saline. NC inhibitor and miR-451 inhibitor were synthesized by Ribobio Co. (Guangzhou, China). The mice in miR-451 inhibitor and its NC inhibitor group were injected with a miR-451 inhibitor (5 nmol/g/day) or the same dosage of a NC inhibitor every other day via the retro-orbital plexus beginning at one day before Dox injection. Seven days after Dox injection, pressure-volume loop analysis was performed. After that, all the mice were sacrificed and heart samples were collected for further assay.

### 2.3. Pressure-Volume Loop Analysis

Left ventricle pressure-volume was analyzed in mice anesthetized with 2% isoflurane by using a 1.0-F pressure-volume catheter (PVR 1045). The position of the catheter was monitored by pressure along with the magnitude and phase using the ARIA pressure-volume conductance system (Millar Instruments, Houston, Texas) and Powerlab A/D converter (AD Instruments, Mountain View, California). During this process, temperature (36.5-37.5°C) and heart rate were constantly monitored.

### 2.4. Isolation of Cardiomyocytes and Mechanics

After being sacrificed, hearts were removed and mounted onto a temperature-controlled Langendorff system. Hearts were digested with Liberase Blendzymes (0.1 mg/ml, Roche Diagnostics, Indianapolis, IN) for 15 min at 37°C. Mechanical assessment was performed in isolated cardiomyocytes within 6 h of isolation. Only rod-shaped myocytes with clear edges were selected for mechanical study. We used an IonOptix™ soft-edge system (IonOptix, Milton, MA) to detect mechanical properties according to a previous study [19]. Cell shortening and relengthening, as reflected by peak shortening (PS) and maximal velocities of shortening/relengthening (± dL/dt), were assessed.

### 2.5. Measurement of NT-proBNP, cTnI, and CK

Blood samples were collected from mice via the retro-orbital plexus at 5 days after Dox injection and centrifuged to separate plasma. Plasma NT-proBNP, cTnI, and CK levels were detected according to the manufacturer's instructions.

### 2.6. Detection of Cardiac 4-HNE, GSH/GSSG, and MDA Levels and Activities of SOD and Caspase3

To detect oxidative stress parameters, the levels of GSH, GSSG, and MDA and the total SOD activity in heart samples were determined according to the manufacturer's instructions using a microplate reader (SpectraMax 190, Molecular Device, USA). Quantitation of 4-HNE-protein adducts was performed using a kit from Abcam under manufacturer's instruction. The activity of casapse3 was detected using a kit obtained from Beyotime Biotechnology (Beijing, China)

### 2.7. Real-Time PCR

We used Trizol reagent to extract total RNA from frozen heart samples. The total RNA was purified using RNeasy columns (Qiagen, Valencia, CA, USA). After that, DNAase-treated RNA was reverse transcribed using PrimeScript1 RT reagent Kit (TaKaRa, Dalian, China) with Bulge-Loop™ miRNA. The quantitation was performed with SYBR Premix Ex TaqTM II (Tli RNaseH Plus, TaKaRa). Expression of the target genes was measured in triplicate and then normalized to *β*-actin.

### 2.8. Western Blot

The total protein from the frozen heart tissues was homogenized using RIPA lysis buffer. Total of 30 *μ*g protein sample was separated on 10% SDS-PAGE gel and then transferred to PVDF membranes [20]. After that, the membranes were incubated with the primary antibodies at 4°C overnight. After reacting with the second antibody, the membranes were stained with enhanced chemiluminescence reagent and scanned by the BIO-RAD ChemiDoc Touch Imaging System (BIO-RAD, Hercules, CA, USA). Densitometric analysis was performed using the Image J software.

### 2.9. TUNEL Staining

The proportion of apoptosis was calculated as the ratio of apoptotic cells to all the cells. The terminal deoxynucleotidyl transferase-mediated dUTP nick end-labelling (TUNEL) was used to detect the apoptosis in the hearts after Dox injection according to the manufacturer's instructions using a commercially available kit (Roche Diagnostics, Mannheim, Germany). In each group, five mice were included. In each mouse, at least 6 fields were counted.

### 2.10. Cell Culture and Treatment

H9c2 cells were cultured in incubated Eagle's minimum essential medium (Sigma Chemical Co., St Louis, MO, USA) supplemented with 10% fetal bovine serum (FBS) and 1% penicillin and streptomycin. After 24 hours, the H9c2 cardiomyocytes were divided into four groups: NC inhibitor+PBS, in which only NC inhibitor (100nmol/l) was added to the cultured medium; miR-451 inhibitor+PBS group, in which only miR-451 inhibitor (100nmol/l) was added; NC inhibitor+Dox, in which NC inhibitor (100nmol/l) was added to the cultured medium and 1 *μ*mol/l Dox was added; miR-451 inhibitor+Dox group, in which miR-451 inhibitor (100nmol/l) and 1 *μ*mol/l Dox were added. To detect cell apoptosis, the H9c2 cells were cultured for 12 hours. To detect the production of 4-HNE and MDA in cardiomyocytes, the H9c2 cells were cultured for 24 hours. MTT assay was used to test the cell viability in our study. Cells were seeded in 96-well plates at 1×10^5^ cells/well, subjected to PBS or Dox and/or miR-451 inhibitor incubated with MTT (5 mg/ml) for 3 h at 37°C. To confirm the role of AMPK, H9c2 cells were treated with compound C (20 *μ*M) in the presence of miR-451 inhibitor and Dox for 12 hours.

miR451-expressing human gastric carcinoma SGC-7901 cells were cultured in RPMI 1640 medium with 10% FBS [21]. After that, SGC-7901 cells were treated with a miR-451 inhibitor (100nmol/l) and Dox (1 *μ*mol/l) for 24 h. After that, cell viability was detected.

### 2.11. ROS Detection

H9c2 cardiomyocytes were cultured in 96-well plates and pretreated with the miR-451 inhibitor or NC inhibitor in the presence of Dox for 6 hours. After that, the cells were incubated with DCFH-DA (10 *μ*mol/l) for 30 min at 37°C to detect the production of reactive oxygen species (ROS), and absorbance was detected by a microplate reader according to a previous study [16].

### 2.12. Statistical Analysis

Data were presented as means+standard deviation. An unpaired t-test was used to compare significance between two groups. One-way ANOVA followed by Student-Newman Keuls's (SNK's) post-hoc test was used to compare difference between two more groups.* P* values < 0.05 were considered to be statistically significant.

## 3. Result

### 3.1. miR-451 Inhibition Attenuated Dox-Induced Cardiac Injury

**Figure 1:**
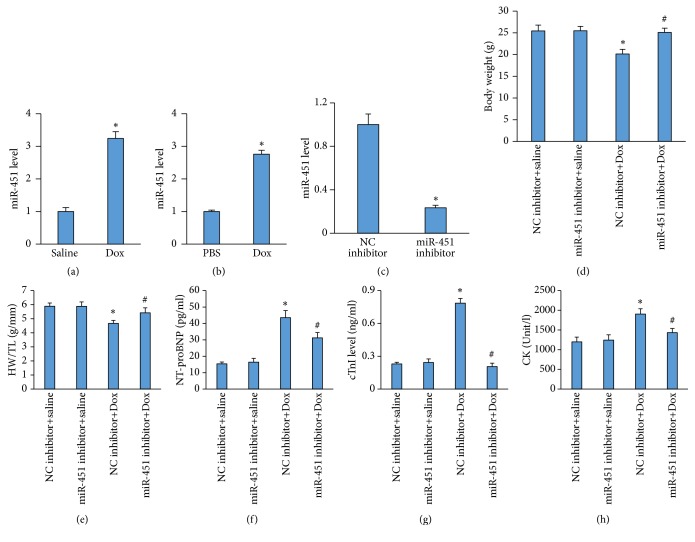
miR-451 inhibition prevented Dox-induced cardiac injury. (a) The level of miR-451 in the hearts (n=5). (b) The level of miR-451 in the H9c2 cells (n=5). (c) The level of miR-451 in the hearts (n=5). (d) Body weight in the Dox-treated mice (n=10). (e) The ratio of heart weight to tibial length in the indicated mice (n=10). (f) The level of NT-proBNP in the hearts (n=6). (g) The level of cardiac cTnI after Dox treatment (n=6). (h) The level of CK in the mice (n=6). *∗P*<0.05 compared with the group with saline or PBS. ^#^*P*<0.05 compared with mice after Dox injection.

The data in our study suggested that miR-451 level was significantly increased in the hearts of Dox-treated mice (Figure 1(a)). To further confirm this finding, H9c2 cells were subjected to Dox for 12 hours. We found that Dox upregulated the expression of miR-451 in vitro (Figure 1(b)). To explore the role of miR-451 in Dox-induced cardiac injury, we used a miR-451 inhibitor to suppress the expression of miR-451 in the hearts (Figure 1(c)). Dox resulted in the decreased body weight, which is a marker of Dox-caused toxic effect. miR-451 inhibition could restore body weight to the normal level (Figure 1(d)). The ratio of heart weight to tibial length was significantly decreased in Dox mice compared to control mice, however markedly recovered in mice with miR-451 inhibition (Figure 1(e)). NT-pro BNP level was significantly higher in mice with Dox injection when compared with that in mice with saline only, which tended to be lower after miR-451 inhibition (Figure 1(f)). Plasma levels of cTnI were effective in evaluating cardiac damage in patients with Dox therapy [22]. The increased cTnI caused by Dox was reduced after miR-451 inhibition (Figure 1(g)). Further detection of plasma CK also revealed that CK was significantly reduced in the mice treated with a miR-451 inhibitor compared with mice treated with the NC inhibitor (Figure 1(h)).

### 3.2. miR-451 Inhibition Improved Dox-Induced Cardiac Dysfunction

**Figure 2:**
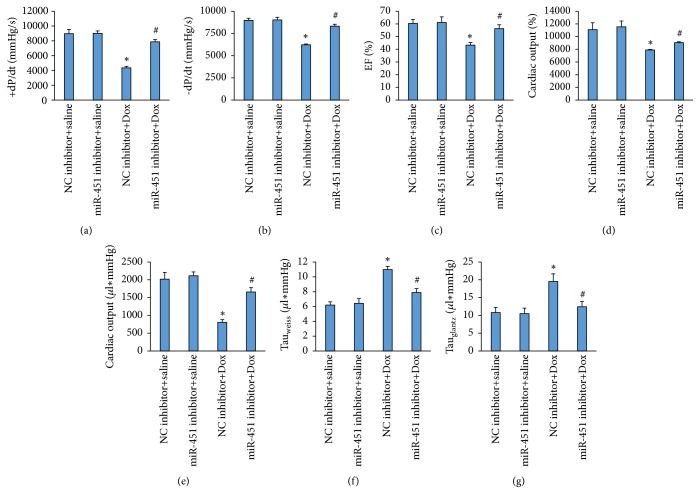
miR-451 inhibition improved cardiac function in mice. (a-b) The alteration in +dP/dt and –dP/dt in mice (n=8). (c) Ejection fraction in the mice (n=8). (d-e) Cardiac output and stroke work in the mice (n=8). (f-g) Tau (Weiss and Glantz) in the mice (n=8). *∗P*<0.05 compared with the group with saline. ^#^*P*<0.05 compared with mice after Dox injection.

Treatment of mice with Dox induced a significant decrease in maximum first derivative of ventricular pressure with respect to time (+dP/dt), -dP/dt, and ejection fraction (EF). miR-451 inhibition significantly attenuated these pathological alterations in Dox-treated mice (Figures 2(a)–2(c)). Cardiac output and stroke volume were significantly increased in the Dox-treated mice with miR-451 inhibition compared with the mice in the Dox+NC inhibitor group (Figures 2(d)-2(e)). Furthermore, prolongation of relaxation time constants (Tau Weiss and Glantz) was increased in the Dox group whereas it reduced after miR-451 inhibitor injection (Figures 2(f)-2(g)).

### 3.3. miR-451 Inhibition Reduced Dox-Induced Cardiomyocyte Contractile Dysfunction

**Figure 3:**
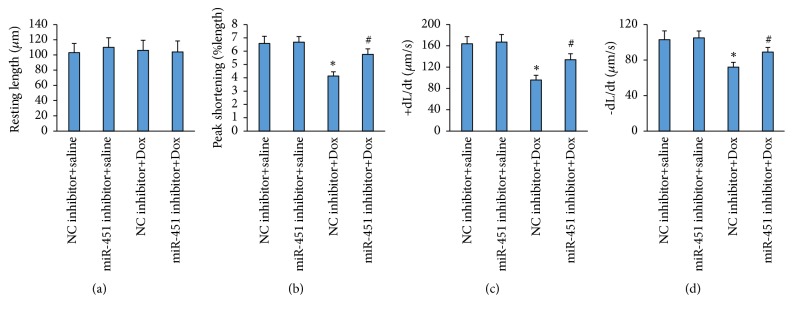
miR-451 inhibition improved cardiomyocyte contractile properties. (a) Resting cell length. (b) Peak shortening (normalized to cell length). (c-d) Maximal velocity of shortening (+dL/dt) and maximal velocity of relengthening (−dL/dt). *∗P*<0.05 compared with the group with saline. ^#^*P*<0.05 compared with mice after Dox injection. n=50 cells from 3 mice per group.

Next, we tried to investigate the effect of miR-451 inhibition on single cardiomyocyte contractile function. There was no difference in resting cell length between four groups (Figure 3(a)). Cardiomyocytes isolated from mice challenged with Dox exhibited a reduced peak shortening and maximal velocity of shortening/relengthening (±dL/dt), all of which were significantly improved in cardiomyocytes isolated from mice treated with Dox+miR-451 inhibitor (Figures 3(b)–3(d)).

### 3.4. Inhibition of miR-451 Could Reduce Oxidative Stress and Cell Apoptosis in Dox-Treated Mice

**Figure 4:**
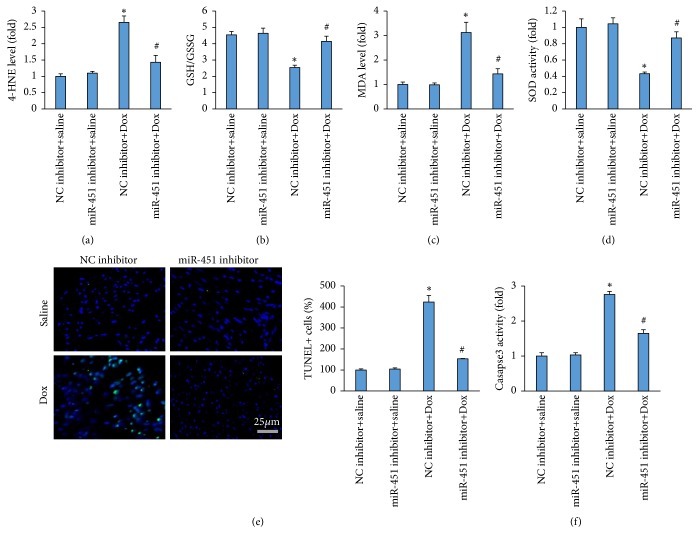
miR-451 inhibition attenuated oxidative stress and apoptosis in mice. (a) The level of 4-HNE in the hearts (n=6). (b) The level of GSH/GSSG in the hearts (n=6). (c) The level of cardiac MDA (n=6). (d) The activity of SOD in the hearts (n=6). (e) Cell apoptosis detected by TUNEL staining (n=5). (f) The activity of caspase3 in the hearts (n=6). *∗P*<0.05 compared with the group with saline. ^#^*P*<0.05 compared with mice after Dox injection.

Dox resulted in the production of quinone and redox-cycling, which promoted ROS-dependent lipid peroxidation and accumulation of 4-HNE [23]. The accumulation of 4-HNE, a cardiac toxic, contributed to cardiac injury and impairment of myocardial contractile function [24]. As shown in Figure 4(a), Dox significantly enhanced 4-HNE accumulation, and conversely this pathological elevation was suppressed after miR-451 inhibition. Inhibition of miR-451 significantly reduced the level of MDA and increased GSH/GSSG and activity of SOD in Dox-treated mice (Figures 4(b)–4(d)). Next, we evaluated the cell apoptosis caused by Dox. Dox notably induced cell apoptosis in the Dox-treated hearts as detected by TUNEL staining, and miR-451 inhibition significantly decreased the proportion of TUNEL positive cells in mice with Dox treatment (Figure 4(e)). Western blot analysis revealed miR-451 inhibition decreased caspase3 activity in the hearts of mice with Dox treatment (Figure 4(f)).

### 3.5. miR-451 Inhibited Attenuated Oxidative Stress and Cell Apoptosis in Dox-Treated H9c2 Cells

**Figure 5:**
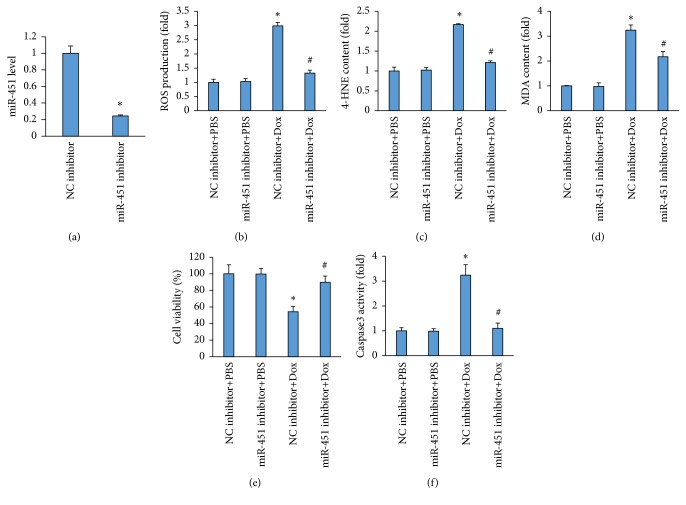
miR-451 inhibition attenuated oxidative stress and apoptosis in H9c2 cells. (a) The level of miR-451 expression after a miR-451 inhibitor treatment (n=6). (b) The level of ROS in H9c2 cells (n=6). (c) The level of 4-HNE in H9c2 cells (n=6). (d) The MDA content in H9c2 cells (n=6). (e) Cell viability after Dox treatment (n=6). (f) The activity of caspase3 in H9c2 cells (n=6). *∗P*<0.05 compared with the group with PBS. ^#^*P*<0.05 compared with Dox-treated H9c2 cells.

To further confirm the role of miR-451 in Dox-induced cell injury, we inhibited miR-451 expression in H9c2 cardiomyocytes in vitro (Figure 5(a)). miR-451 inhibition attenuated the production of ROS in Dox-treated cells (Figure 5(b)). The increased 4-HNE content and MDA level in cells with Dox treatment were also suppressed after miR-451 inhibition (Figures 5(c)-5(d)). Next, we evaluated miR-451 inhibition on cell loss induced by Dox, and found that miR-451 inhibition significantly improved cell viability in Dox-treated cells (Figure 5(e)). Further detection of casapse3 activity also revealed that miR-451 inhibition attenuated the upregulation of caspase3 in Dox-treated cells (Figure 5(f)).

### 3.6. miR-451 Regulated AMPK*α* in Mice

**Figure 6:**
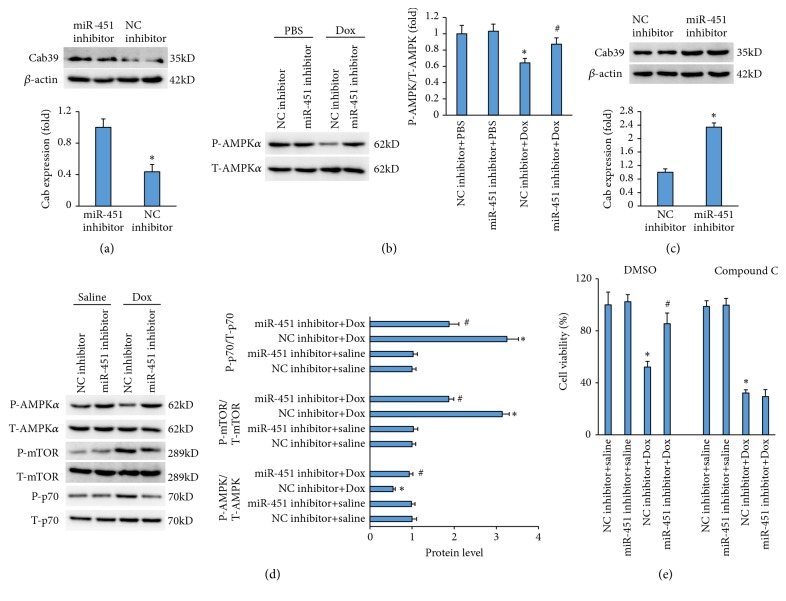
miR-451 inhibition provided cardioprotection via activating AMPK signaling pathway. (a) The level of Cab39 in the H9c2 cells (n=6). (b) AMPK signaling pathway in the H9c2 cells (n=6). (c) The level of Cab39 in the hearts (n=6). (d) AMPK signaling pathway in the hearts (n=6). (e) Cell viability in H9c2 cells (n=6). *∗P*<0.05 compared with the group with PBS or saline. ^#^*P*<0.05 compared with Dox-treated H9c2 cells or hearts.

A previous study reported that miR-451 directly interacted with the 3'-UTRs of Cab39 [12]. Therefore, we first detected the Cab39 protein levels and found that miR-451 knockdown increased Cab39 protein level in H9c2 cells (Figure 6(a)). Cab39, an armadillo repeat scaffolding-like protein, is a component of the trimeric liver kinase B1 (LKB1)-STRAD-Cab39 complex and stabilizes the activity of LKB1 [13, 14]. It has been reported that LKB1 complexed to Cab39 activated AMPK by phosphorylating Thr172 and that Cab39 increased phosphorylation of AMPK up to about 100-fold. Next, we detected AMPK using immunoblotting. Suppression of AMPK phosphorylation was observed in H9c2 cells with Dox treatment, and miR-451 inhibition significantly increased the phosphorylation of AMPK*α* (Figure 6(b)). Western blotting analysis was performed to determine the AMPK*α* pathways in the hearts. As expected, we also found that Cab39 expression was significantly increased in the mice with miR-451 inhibition compared with the mice with NC inhibitor (Figure 6(c)). AMPK*α* phosphorylation at Thr172 was also significantly enhanced in the hearts of mice with miR-451 inhibition compared with mice with NC inhibitor after Dox treatment (Figure 6(d)). The phosphorylation of mTOR and p70, the downstream targets of AMPK*α*, which were significantly increased in mice with Dox+NC inhibitor, was prevented by miR-451 inhibition (Figure 6(d)). To further verify the role of AMPK*α*, we used compound C, which is an antagonist of AMPK*α*, for further experiment and found that the protection provided by miR-451 inhibition on cell viability was abolished after compound C treatment (Figure 6(e)).

### 3.7. miR-451 Inhibition Does Not Affect Dox-Induced Cell Death in Gastric Cancer Cells

**Figure 7:**
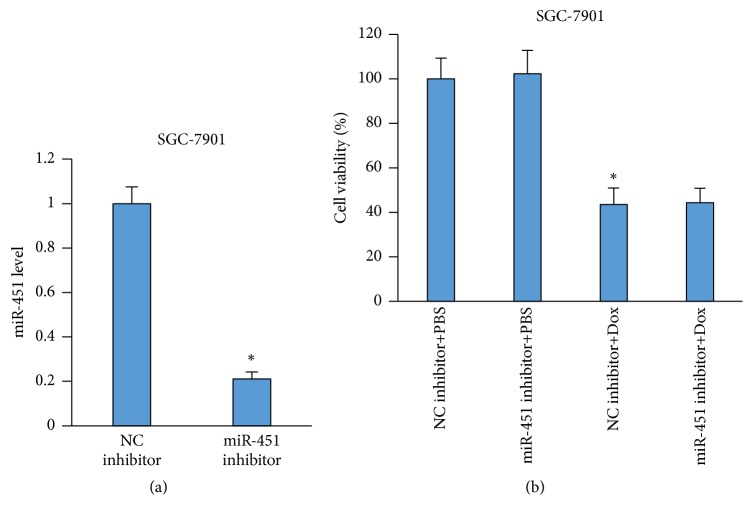
miR-451 inhibition cannot affect Dox-induced death of cancer cells. (a) The level of miR-451 expression after a miR-451 inhibitor treatment (n=6). (b) Cell viability (n=6). *∗P*<0.05 compared with the group with NC inhibitor.

To determine whether miR-451 inhibition could attenuate the death of cancer cells, we used miR451-expressing human gastric carcinoma SGC-7901 cells and found that miR-451 inhibition did not compromise the oncological efficacy of Dox in gastric carcinoma cells (Figures 7(a)-7(b)).

## 4. Discussion

A previous report demonstrated that miR-451 had a high expression in the hearts, and miR-451 mainly located in the cardiac myocytes [12]. Although miR-451 has been shown to play a key role in the pathogenesis of diabetic cardiomyopathy in obese mice [12], miR-451 has not been further investigated for additional function in the hearts, especially in Dox-induced cardiac injury. To the best of our knowledge, this is the first report describing that miR-451 inhibition produced a protective effect against Dox-induced cardiotoxicity in mice and in H9c2 cells. In this study, miR-451 inhibition also attenuated Dox-induced production of oxidative stress and cardiomyocytes apoptosis in vivo and in vitro. Moreover, inhibition of miR-451 could improve Dox-induced cardiac dysfunction in the mice and reduced Dox-induced cardiomyocyte contractile dysfunction. We also found that miR-451 inhibition resulted in the increased expression of Cab39 and enhanced activation of AMPK*α* signaling pathway in vivo and in vitro. These findings positively suggest the potential clinical translation of the miR-451 inhibitor into a therapeutic drug for pathological cardiac injury.

Initial study found that miR-451 expression was mainly expressed in erythropoietic cells [10]. miR-451 deficiency could lead to the impairment of erythrocyte differentiation [11]. Recently, several studies have noted the association between miR-451 and cardiovascular diseases. A study found that miR-451 was also highly expressed in cardiac myocytes, and miR-451 could promote cardiac hypertrophy in obese mice [12]. Song et al. found that miR-451 was decreased in the hearts of patients with hypertrophic cardiomyopathy [25]. However, there sounds a quite different voice that cardiac miR-451 was found to be increased in response to high-fat diet or ischemic preconditioning [12, 26]. Consistent with these studies, we also found that miR-451 expression was increased in the hearts collected from mice with Dox treatment or Dox-treated cardiomyocytes. These findings prompted us to investigate whether optimising miR-451 levels in Dox-treated hearts could prevent Dox-related cardiac injury. miR-451 inhibition significantly attenuated Dox-induced cardiac injury, as reflected by the HW/TL, the levels of NT-proBNP, and cTnI. miR-451 inhibition also restored the cardiac function in Dox-treated mice and improved cardiomyocyte contractile function. However, these data cannot be overinterpreted for the reason that there is limited and conflicting information about the role of miR-451 in cardiovascular diseases. The results come from Fan's lab showed that loss of the miR-144/451 cluster impaired ischemic preconditioning-mediated cardioprotection [26]. Song et al. found that the decrease of miR-451 might contribute to the development of hypertrophic cardiomyopathy [25]. The two studies were incompatible with our finding that miR-451 inhibition provided cardioprotection, which has been confirmed by a previous report using cardiomyocyte-specific miR-451 knockout mice [12]. These conflicting results might be explained by different diseases and different approaches to inhibit miR-451.

The hearts had a lot of mitochondria and relative lower levels of antioxidant enzymes when compared with other organs, rendering the heart particularly vulnerable to free radical damage and Dox-induced cardiotoxicity [27]. Oxidative stress could be observed in heart samples within three hours after Dox treatment [28]. Several studies have suggested that oxidative stress played critical role in the pathogenesis of Dox-induced cardiac damage [18, 27]. ROS produced during Dox treatment caused lipid peroxidation and structural changes of biological macromolecules and ultimately resulted in cell death [1]. In our model, multiple lines of evidence suggested that Dox induced the production of oxidative stress in cardiac tissues, and miR-451 inhibition exhibited the capacity to suppress oxidative stress induced by Dox. Cardiomyocyte apoptosis might be the leading cause of cardiac dysfunction in Dox-induced cardiomyopathy. Consistent with this, the results indicated that Dox injection resulted in cell loss, whereas miR-451 inhibition almost abolished these pathological alterations.

The data in our study showed that miR-451 knockdown provided protection against Dox-related cardiac injury. To clarify the direct targets of miR-451, we focused on Cab39. Our results found that miR-451 inhibition increased the expression of Cab39. Cab39 can stabilize the activity of LKB1 and thus activate AMPK*α* signaling pathway [13, 14]. As expected, we also found that miR-451 inhibition enhanced the phosphorylation of AMPK*α* and suppressed the activation of mTOR and p70. AMPK*α* has been shown to suppress redox imbalance and cell death in the rats [17]. Moreover, activation of AMPK*α* by molecules could provide cardioprotection against Dox-induced toxicity [18, 29, 30]. Next, we tested whether AMPK*α* was involved in the protection of miR-451 inhibition using Compound C. We found that compound C completely offset the protective effect of miR-451 knockdown in cell viability, implying that miR-451 inhibition exerted its function via activating AMPK*α*. It has been reported that cardioprotective effect of metformin against Dox-induced toxicity is mediated via upregulation of AMPK [31]. However, high doses of metformin treatment lost its protective effect on Dox-induced toxicity due to suppress of platelet-derived growth factor receptor [31]. A recent study demonstrated a link between metformin use and early onset lung cancer [32]. These finding might compromise the translational potential of metformin in Dox-related injury.

It has been reported that miR-451 was expressed in human non-small cell lung cancer and played a role in acquiring chemoresistance through upregulating multidrug resistant protein-1[33]. It was important to confirm that miR-451 inhibition does not compromise the oncological efficacy of Dox. In our study, we found that miR-451 inhibition cannot affect Dox-induced cell death of human gastric carcinoma SGC-7901 cells. Our findings thus encourage further studies on the translational potential of the miR-451 inhibitor.

In conclusion, miR-451 inhibition induced the phosphorylation of AMPK*α* and partially inhibited Dox-induced oxidative stress and cell apoptosis in cardiomyocytes. We suggest that miR-451 inhibitor is protective in DOX-induced cardiomyopathy through AMPK signaling pathway. miR-451 inhibition could provide a novel therapeutic possibility for the prevention of Dox-induced cardiotoxicity in cancer patients.

## Data Availability

The data are available from the corresponding author upon reasonable request.
